# Quantification of Construction Materials Quality via Frequency Response Measurements: A Mobile Testing Station

**DOI:** 10.3390/s23218884

**Published:** 2023-11-01

**Authors:** Lukasz Scislo, Nina Szczepanik-Scislo

**Affiliations:** 1 Faculty of Electrical and Computer Engineering, Cracow University of Technology, Warszawska 24, 31-155 Cracow, Poland; 2 Faculty of Environmental Engineering and Energy, Cracow University of Technology, Warszawska 24, 31-155 Cracow, Poland; nina.szczepanik@pk.edu.pl

**Keywords:** vibration measurements, impulse excitation technique, material testing, mechanical properties, frequency response

## Abstract

In construction, ensuring the quality and compliance of materials with specified requirements is often challenging, especially at construction sites. Conventionally, this process necessitates transporting samples to well-equipped laboratories, incurring significant time and financial costs. This article proposes a novel approach through a cost-effective mobile test station, enabling on-site measurements and immediate evaluation results, regardless of the testing conditions. The foundation of our testing methodology lies in the Impulse Excitation Technique (IET), which capitalises on measuring the frequency response of samples while considering their mass and dimensions. By applying this technique, we can effectively determine crucial elastic properties, such as the Young Modulus and Poisson Ratio. These obtained values can then be cross-referenced with established material tables to verify the material’s compliance with the specified order. In this study, the developed universal and mobile test station demonstrated versatility by successfully evaluating three samples of typical construction materials, showing the method’s reliability on some real case measurements. The results substantiate its potential as a reliable mobile quality assurance station. Moreover, the station’s adaptability empowers its use on site, in laboratory settings, or even during material transportation when necessary. This innovation promises to revolutionise material quality assessment, streamlining the construction process and expediting decision making.

## 1. Introduction

Construction materials are used to build machines and devices but also in civil engineering for construction purposes [1]. They are mainly metals and their alloys, plastics, ceramics, and composites. The construction materials used, e.g., for supports and creating connections of different elements, can be divided into metallic (metals and their alloys), non-metallic, and composite materials. Material selection depends on the working conditions in which the materials are used. It is imperative that when creating new or producing typical construction products, their specific properties are known.

Due to the wide range of applications, metallic structures are most commonly used. Typical metals used in civil engineering include:

- Steel: used in the construction of buildings, bridges, and infrastructure due to its strength and durability.
- Aluminium: lightweight, corrosion-resistant, and strong, making it suitable for use in construction, transportation, and infrastructure.
- Copper: commonly used in electrical wiring, plumbing, roofing, and flashing.
- Iron: used in reinforcing concrete, making structural beams, and casting iron pipes.
- Lead: used in roofing, flashing, and radiation shielding.
- Zinc: used for galvanizing steel to protect it from corrosion and for roofing and flashing.

These metals are chosen for their specific properties, such as strength, durability, and corrosion resistance, making them suitable for various civil engineering applications.

The quality control of metals in civil engineering involves various steps to ensure that the materials used in construction meet the required specifications and standards. Some common methods include the following:

- Visual Inspection: This step requires checking the metal for cracks, corrosion, or other visible defects in construction, structures, and components. This, the most classical approach presented in numerous works [2,3], has been extensively studied in recent years due to the incorporation of many novel optical techniques and algorithms. This includes the use of high-resolution optical devices in connection with specialized algorithms, e.g., neural networks and deep learning [4,5], drones and other Unmanned Aerial Vehicles as inspection agents [6,7], motion amplification cameras or video stream analysis [8,9], and many more.
- Non-Destructive Testing (NDT): This is one of the most common techniques successfully used to inspect building materials and uses sonic and ultrasonic testing based on elastic wave propagation [10]. Another possibility is radiography or tomography techniques, which are especially useful for the discovery of internal defects [11,12]. Similarly, to detect a hidden defect, a magnetic particle inspection technique can be applied [13,14]. This technique, used for ferromagnetic materials (alloys), allows for detecting shallow surface errors and discontinuities.
- Chemical Analysis: This is used to analyse the chemical composition of a metal to verify that it meets the required specifications [15,16]. However, this technique is very accurate, requiring specialized equipment and time to perform tests and analyses.
- Mechanical Testing: This is used to test the metal’s strength, ductility, and hardness to verify that it meets the designer’s needs or can be applied in specific solutions [17]. However, this technique is considered destructive (test sample requirements) and allows for the use of tiny test objects [18].
- Corrosion Testing: This technique is generally based on exposing the metal to a simulated environment to test its corrosion resistance. The data acquired are especially useful for predicting the corrosion effect of structures in an urban environment [19].
- Fatigue Testing: This generally subjects the metal to repeated loading and unloading cycles to assess its ability to withstand repeated stress. This information is precious as input for simulation software (e.g., in the case of new materials) and in the case of high-rise building designs [20,21,22].
- Environmental Testing: This technique is based on exposing the material to extreme environmental conditions, like low and high temperature, humidity, and other environmental conditions, to test its performance [23,24].

**Figure 1:**
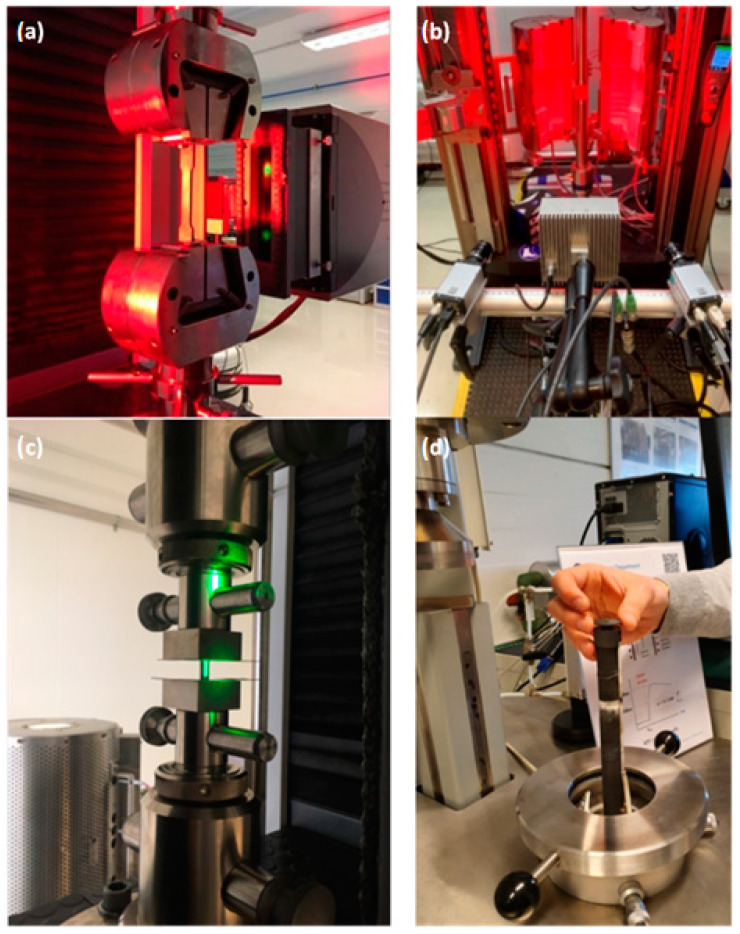
Typical material tests performed in laboratory conditions: (**a**) mechanical testing (tensile testing); (**b**) digital image correlation; (**c**) compression test; (**d**) environmental testing and thermo-physical analysis.

Regardless of the chosen method, these tests aim to ensure that the metal meets the required quality standards, is suitable for its intended use, and will perform as expected over time. Figure 1 shows some of the typical material testing solutions in laboratory conditions at the Mechanical Measurement Laboratory at CERN, where various materials must be tested to verify their data (input for finite element analysis (FEM)) and their usefulness for particle detectors and supporting structures and systems.

Most of these tests are performed in laboratory conditions to provide a controlled environment for accurate and repeatable results. The results of these tests are then used to verify that the metal meets the required specifications and quality standards and to determine its suitability for its intended use in civil engineering applications. Additionally, reliable material parameters are used as input values for computer simulations (e.g., finite element method simulation) to decrease the eventual error due to inaccurate material assumptions. Most techniques’ drawbacks are the need for expensive equipment (e.g., a tomograph or universal testing machine), access to adequately equipped laboratories, and, especially, well-trained test engineers. Thus, professional material testing requires sufficient resources (equipment or money) and testing and data evaluation time.

However, in the case of on-site quality control, where access to specialised equipment is problematic and time for the test is limited, the options are also limited and usually rely on visual inspection (visible defect identification) and dimensional inspection (assurance that dimensions and tolerances are met).

This paper aims to present a tool, testing station design, and the methodology for the on-site quick evaluation of material properties without expensive and time-consuming laboratory tests. These on-site testing stations and testing algorithms based on the Impulse Excitation Technique (IET) are designed to ensure that the metal meets the required quality standards and, when installed, will perform as designed over time. The results of this procedure are documented and verified on several typical construction material samples and show that typical visual and dimensional inspection can be extended by fast on-site measurement, where the whole procedure, including setup, test, and data evaluation, lasts only a few minutes. Additionally, with the Industry 4.0 concept, the data can be transferred immediately after the test to the cloud for additional processing, calculations, or simple historical data analyses.

## 2. Materials and Methods

### 2.1. Material Testing Based on Frequency Response Analysis

Frequency Response Function (FRF) is a commonly used term in material testing and refers to the relationship between a system’s input and output signals, usually expressed in the frequency domain. In material testing, FRFs are used to study the dynamic behaviour of materials and structures. FRFs are typically obtained by applying a known input excitation, such as a sinusoidal wave or a step function, to the material or structure and measuring the resulting response [25,26]. The input and output signals are then transformed from the time domain into the frequency domain using techniques, such as Fourier analysis, allowing for a more detailed examination of the system’s behaviour over a range of frequencies. The FRF can be used to determine important information about the system, such as its natural frequencies, damping ratios, and mode shapes, which can be used to optimize the design and performance of the material or structure. It can also be used to identify structural weaknesses or anomalies, such as cracks, loose fasteners, or other types of damage. Overall, the FRF is a powerful tool in material testing and is widely used in civil engineering, mechanical engineering, aerospace engineering, and other fields to study the dynamic behaviour of materials and structures.

One of the possibilities of using the FRF for construction material testing is the so-called Impulse Excitation Technique (IET). The Impulse Excitation Technique is used in material testing to study the dynamic behaviour of materials and structures [27,28]. The principle behind the Impulse Excitation Technique is to apply a sudden, high-energy impulse to the material or structure and measure its resulting response. This impulse can be generated using various methods, such as a hammer, a drop weight, or a shaker. Once the impulse is applied, the resulting response of the material or structure is typically measured using accelerometers or other types of sensors. The signals from these sensors are then analysed to determine the system’s frequency response, which can provide information about its dynamic behaviour, such as its natural frequencies, damping ratios, and mode shapes. The Impulse Excitation Technique helps researchers to study the behaviour of materials and structures under dynamic loads, such as during earthquakes, impacts, or other high-energy events [28,29]. By understanding the dynamic behaviour of these materials and structures, engineers can design and build structures that are more resistant to these loads and provide improved safety and performance. Another use is a non-contact sensor (e.g., microphone, Laser Doppler Vibrometer [30]) and a well-known procedure to calculate some material properties, like dynamic Young Modulus, dynamic shear modulus, or Poisson’s Ratio.

### 2.2. IET Testing Principles and Calculations of Material Elastic Properties

**Figure 2:**
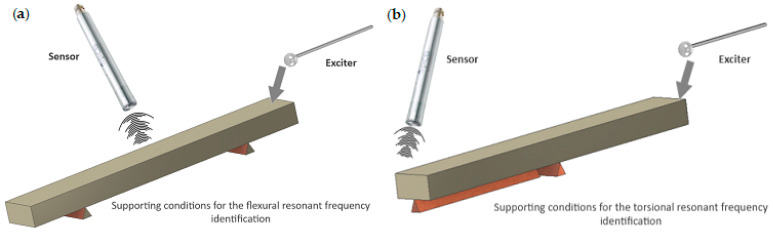
IET procedure schema: (**a**) obtaining the first flexural mode; (**b**) obtaining the first torsional mode.

Figure 2 presents the testing conditions for obtaining Flexural Resonant Frequency and Torsional Resonant Frequency. The specific orientation of the supports, sensor, and the exciter is presented. It is also possible to exchange the supports and hang the samples on elastic lines in a free-free condition.

Acquiring the dynamic Young’s Modulus requires calculations based on the detected first flexural mode, sample mass, and dimensions (Equation (1)).

(1) $$E=0.9465\;(\frac{m\;f_{f}^{2}}{b})(\frac{L^{3}}{t^{3}})T_{1}$$

where

E—Young’s Modulus, Pa
m—mass of the sample (a bar), g
b—width of the sample (a bar), mm
L—length of the sample (a bar), mm
t—thickness of the sample (a bar), mm
$f_{f}^{}$—fundamental resonant frequency of sample (a bar) in flexure, Hz
T_1_—correction factor for fundamental flexural mode to account for finite thickness of the sample (a bar), Poisson’s ratio, and so forth.

Additionally, if $\frac{L}{t}\geq 20$, $T_{1}$ can be simplified into the following

(2) $$T_{1}=[1.000+6.585{\left(\frac{t}{L}\right)}^{2}]$$

Similarly, the dynamic Shear Modulus can be calculated after acquiring the first torsional mode using Equation (3).

(3) $$G=\frac{4Lm\;f_{t}^{2}}{bt}R$$

where

G—dynamic shear modulus, Pa,
$f_{t}^{}$—fundamental resonant frequency of sample (a bar) in torsion Hz.

(4) $$R=\left[\frac{1+{\left(\frac{b}{t}\right)}^{2}}{4-2.521\frac{t}{b}(1-\frac{1.991}{e^{\pi \frac{b}{t}}+\;1})}\right]\left[1+\frac{0.00851b^{2}}{L^{2}}\right]-0.060{\left(\frac{b}{L}\right)}^{\frac{3}{2}}{\left(\frac{b}{t}-1\right)}^{2}$$

Finally, Poisson’s ratio can be simply acquired after using Equation (5).

(5) $$\mu =\frac{E}{2G}-1$$

### 2.3. Mobile Testing Station Prototype for On-Site Impulse Excitation Testing

**Figure 3:**
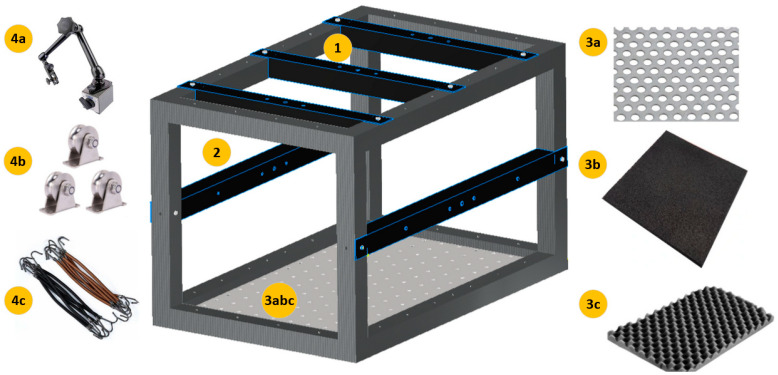
IET testing station design: 1-removable top elements; 2-removable side elements; 3a-bottom sheet metal plate; 3b-layer of a rubber plate; 3c-layer of convoluted foam; 4a-magnetic foot; 4b-line pulleys; 4c-flexible lines.

For the incorporation of the testing principle in the construction site environment, a mobile test station was designed (Figure 3). The design concept was based on the following needs:

- The test station is mobile. Thus, the total weight must be limited so a single person can move it and perform the required tests;
- The test station needs to allow for the testing of tiny samples but also larger ones. Thus, the side and top elements are removable and can be connected in different places or heights (Figure 3 in black-colour elements 1 and 2). Additionally, the holes in each element allow for mounting line pulleys (4b) or flexible lines (4c) for suspending elements in free-free conditions;
- The bottom of the test station (3abc) allows for different tests depending on the users’ needs. It consists of a perforated sheet metal plate (3a) that allows for fixing elements with screws, a layer of a rubber plate (3b) to isolate the testing area from environmental conditions (e.g., ground motion transfer), and a layer of convoluted foam (3c) for testing lightweight materials in near free-free supporting conditions.
- The magnetic feet (4a) allow for fixing sensors or additional elements.

It must be pointed out that IET is a non-destructive, non-contact technique. The purpose of using non-contact methods is usually to not add mass to the testing sample and, thus, not change its dynamic conditions. In consequence, only reliable data are acquired from the sample.

### 2.4. Materials Used for Mobile Station Testing

To test the mobile station, three typical samples of materials used in civil engineering applications were examined:

- Structural steel (S235 according to European Standard EN 10025 or A283C according to the American Society for Testing and Materials (ASTM)). Due to its universality, structural steel S355 is used to manufacture many of the constructions being created for various purposes. They are used as quality steels with guaranteed parameters sufficient in significant industrial installation applications, from building construction and drilling rigs to pipelines distributing media under increased pressure. This steel is universal due to its usage and suitability for welding. The physical properties of structural steel (EN S235) are presented in Table 1.

- Aluminium alloy (EN AW-2017A or ASTM 2017). The 2017 aluminium alloy is characterized by good strength properties and high tensile and fatigue strength. It is suitable for welding and moderately resistant to corrosion. It is used in the production of structural elements. The physical properties of aluminium alloy (EN AW-2017A) are presented in Table 2.

- Brass alloy (EN CW617N or ASTM C37800). This alloy is characterized by good susceptibility to machining. It is widely used in the production of the forged parts in complex shapes, parts for pipes, industrial clamps, heating elements, water and sewage systems, and industrial fittings. The physical properties of a brass alloy (EN CW617N) are presented in Table 3.

The samples were chosen due to knowledge of the properties of the material given by the manufacturers, allowing one to check the calculated values using the method described in this article and connect them to the values given by the manufacturers. Additionally, all three samples were used to calibrate vibration measurement systems and show some of the typical measurement solutions at CERN Mechanical Measurement Laboratory. One of the methods tested for evaluation is the use of 3D Laser Doppler Vibrometry.

It must be pointed out that the IET method is extensively evaluated and, due to its reliability, incorporated in the norms. For rectangular bars, cylinders in longitudinal mode (ASTM E 1876-15), for discs (ASTM E 1876-15), for coatings (ISO 20343), and additional shapes, like, e.g., grinding discs, pipes, etc., which are not included in the norms but can be found in the literature. Thus, it may be used to test most of the typical shapes of the supplied materials before use in the machining of different parts. The method is also easily applicable to different, even low-cost, measuring solutions like e.g., mobile device sensors [31].

## 3. Results

**Figure 4:**
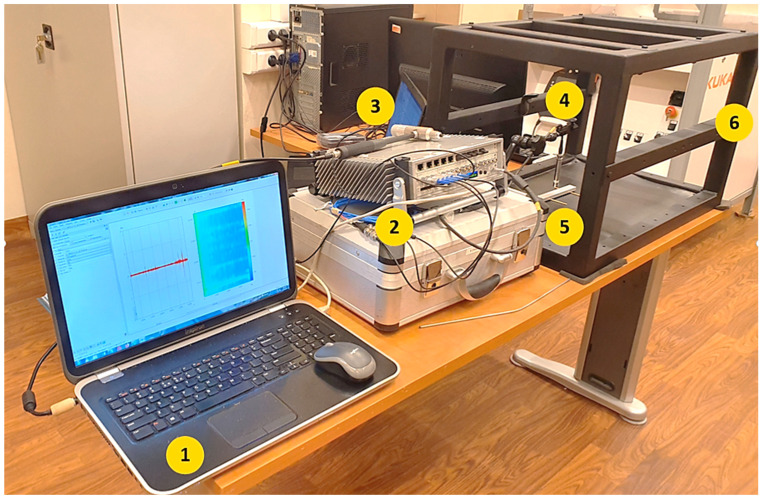
Test setup overview.

An experiment was conducted on three different material samples to verify the procedure and the test station as a universal tool for performing quick on-site tests. The apparatus suitable for accurately detecting, analysing, and measuring a vibrating free-free beam’s fundamental resonant frequency or period was also used. The test apparatus is shown in Figure 4.

It consists of a frequency readout device made up of a computer with specific software (1), an electronic system consisting of a signal conditioner/amplifier, a signal analyser (2), a force impulse generator (3), a suitable transducer to convert the mechanical vibration into an electrical signal (4), and a support system, which, in this case, is the tested mobile station (6). Commercial instrumentation is available that measures the frequency or period of the vibrating specimen (5). The specific, state-of-the-art analyzer MK II and PAK 5.11 software (from Müller-BBM VibroAkustik Systeme GmbH) are used for signal acquisition and response analysis. However, many different solutions are available on the market, including mobile, digital ICP–USB signal conditioners, and, e.g., MATLAB or LabView software. This solution further reduces the test equipment needed for on-site measurements.

The specimens shall be prepared to be rectangular or circular in cross-section. Either geometry can be used to measure both the dynamic Young’s Modulus and dynamic Shear Modulus. Although the equations for computing Shear Modulus with a cylindrical specimen are simpler and more accurate than those used with a rectangular bar, experimental difficulties in obtaining Torsional Resonant Frequencies for a cylindrical specimen usually preclude its use for determining Shear Modulus.

**Figure 5:**
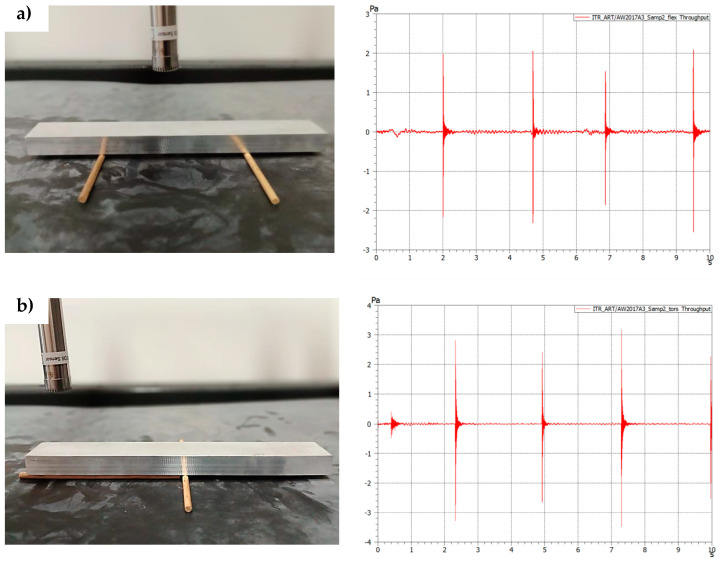
Experimental test on the aluminium sample (sample 2) (**a**) support setup for flexural frequencies excitation together with response graph in time domain; (**b**) support setup for torsional frequencies excitation together with response graph in time domain.

**Figure 6:**
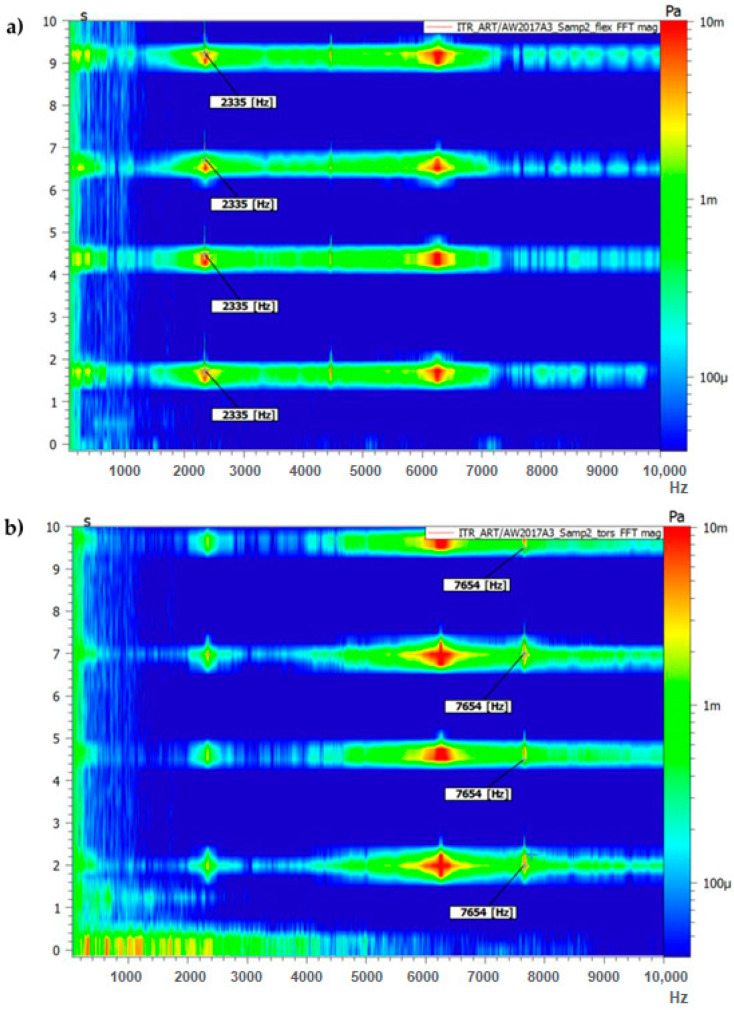
Results of frequency response analysis (FFT magnitude) on the aluminium sample (sample 3): (**a**) support setup for flexural frequencies excitation; (**b**) support setup for torsional frequencies excitation.

Figure 5 and Figure 6 present the results of the measurement performed on the aluminium (EN AW-2017A) samples (first 10 s) for the support setup for excitation of the flexural forms (a) and torsional frequencies (b).

The force input signals visible in Figure 5 correspond to the response in the frequency domain visible in Figure 6.

As can be seen from Figure 5 and Figure 6 after even a low-power impact is applied to a sample, there is an immediate response visible in the frequency domain. Multiple studies confirm that the impact force applied to the test object does not affect the measurement itself [27,32].

However, it is worth mentioning that there are two ways to excite the sample, manually or automatically. In laboratory conditions, an automated modal hammer can be applied. This solution is, however, more suitable for laboratory conditions than the quick, low-cost solution for on-site measurements presented in this paper. In the case of using manual excitation with a handheld hammer, the main problem is that the impact applied to the sample must be of a ballistic type (point contact). The other problem is not using multiple force inputs during the initial excitation (multi-tapings) or tapping before the previous excitation energy is dissipated. The first problem is solved with the unique design of the manual impact hammer with the ball element as a tip. The second problem can be solved while observing the time-domain graph online (Figure 5) and applying another impulse when the previous energy is entirely dissipated. Both of those elements were applied in this study.

To obtain the first flexural mode of the sample, it was placed on the supports, as shown in Figure 2a. Several force impulses were applied, which are visible in Figure 5a and are associated with the frequency responses visible in Figure 6a.

For torsional mode excitation, the supporting system was changed to the one allowing us to obtain the first torsional mode (Figure 2b). In consequence, force impulses were applied (Figure 5b), and the first torsional frequency can be clearly identified in Figure 6b as the frequency that was not visible while the sample was supported in the previous setup).

To obtain a proper statistical evaluation of the systems, for each of the samples (S1–S3), three measurements were performed with three response measurement results taken from each test. An example of one of those measurements is presented in Figure 5 and Figure 6. Then, those three results were averaged and used to calculate mechanical properties. Finally, the three averaged results from three separate tests were placed in Table 4 and averaged again to receive the final result for mechanical properties.

Table 4 presents the averaged data from multiple measurements on each of the three samples from the same material. As is visible, for each analysed sample, a comparison of three tests shows the good reliability and repeatability of the system and method presented. In the case of each material sample response analysis, the impact on the Flexural Resonant Frequency and the Torsional Resonant was in the range of just a few Hz. Slight differences were probably due to some minimal differences in the sample preparation process or minimal differences, e.g., in the weight or dimensions (manufacturing with ±0.01 mm dimensional tolerance). This is also why, for statistical reasons, it was decided to calculate the approximated values of material properties (Table 4). It must be pointed out that the final value corresponds well with the data of material properties given by the manufacturers (Section 2.2). The final comparison is presented in Table 5.

As can be seen, the correlation between the experimental values obtained on the designed mobile testing station and the values given by the manufacturers is in the same range. Keeping in mind that the aim of this study was to perform quick quality checks on any test site, the values show very good method reliability.

## 4. Conclusions

Many techniques can be used to obtain basic material properties, like Young’s Modulus, Shear Modulus, or Poisson’s Ratio. Those techniques can be destructive, e.g., tensile testing using the universal testing machine, or non-destructive, e.g., using a Digital Image Correlation setup. In both cases, expensive equipment and specialized personnel are required.

Alternatively, the IET method is a fast way to obtain frequency response data using simple calculations to acquire material properties, taking just a few seconds. However, the method has some limitations that must be considered for specific field tests.

The most important limitation is that the IET method is specifically designed to obtain the properties data of samples that are elastic, homogeneous, and isotropic. Nevertheless, this dynamic testing procedure has numerous advantages over typical laboratory experiments like static loading techniques. It is also more profitable than ordinary resonant techniques due to not requiring the application of continuous excitation.

It must also be noted that this method belongs to non-destructive tests. For practical purposes, the samples may have a larger structure (e.g., beam used for construction) or small samples obtained from the larger specimen or even the ones already prepared for other tests. Moreover, the crucial advantage is in the cost minimalization, where there is no necessity to use expensive machinery for the test but also no need for a complicated supporting system or setup. The IET requires only stable measurement conditions (e.g., using the presented experimental setup), an impact tool, and a set of simple supports or elastic strings.

Although, in this evaluation, the samples had the regular shape of a beam, which is the most common and, from a practical point of view, the easiest to test, the method can be used for testing both regular and complex shapes. There are numerous procedures for obtaining the natural frequencies of samples with a particular geometry.

The proposed test station was used to test different material samples to validate its usefulness. The results used for calculating Young’s modulus, Shear Modulus, or Poisson’s Ratio match the values of those parameters for the specific alloy. Additionally, as is visible in Figure 5 and Figure 6, the whole testing procedure of one sample, including changing support conditions, takes less than a minute. The results of the frequencies can be read straight from the graphical output or from a data sheet export file. Additionally, to automate the calculations, a Phyton script was prepared. Thus, the goal of creating a fast method to calculate material properties and check the values with the order description was achieved.

Finally, it is also worth mentioning that the IET method is not only used for material characterization and quality control but also for new material development, especially designing data generation. In this last case, unknown materials can be tested to obtain material properties for FEM simulations to improve their accuracy.

## Figures and Tables

**Table 1:** Physical properties of structural steel (EN S235).

Density [kg/cm^3^]	Electr. Conductivity [MS/m]	Thermal Conductivity [W/(mK)]	Modulus of Elasticity [GPa]	Shear Modulus [Gpa]	Poisson’s Ratio	Specific Heat [J/(kg·K)]
7800	≈12	40–45 *	205–2010 *	80–82 *	0.25–0.30 *	460–480 *

* Depending on the manufacturer, the alloy composition, heat treatment, the presence of impurities, and the manufacturing standard used.

**Table 2:** Physical properties of aluminium alloy (EN AW-2017A).

Density [kg/m^3^]	Electr. Conductivity [MS/m]	Thermal Conductivity [W/(mK)]	Modulus of Elasticity [GPa]	Shear Modulus [GPa]	Poisson’s Ratio	Specific Heat [J/(kg·K)]
2790	≈28	130–200 *	72–74 *	27–28 *	0.34–0.37 *	840–860 *

* Depending on the manufacturer, the alloy composition, heat treatment, the presence of impurities, and the manufacturing standard used.

**Table 3:** Physical properties of a brass alloy (EN CW617N).

Density [kg/m^3^]	Electr. Conductivity [MS/m]	Thermal Conductivity [W/(mK)]	Modulus of Elasticity [GPa]	Shear Modulus [GPa]	Poisson’s Ratio	Specific Heat [J/(kg·K)]
8430	≈15	110–120	95–125 *	35–44 *	0.34–0.40 *	≈377 *

* Depending on the manufacturer, the alloy composition, heat treatment, the presence of impurities, and the manufacturing standard used.

**Table 4:** IET measurement and calculation results.

Sample/ Measurement Number	Flexural Resonant $\mathrm{Frequency}\;f_{f}^{}$ [kHz]	Torsional Resonant $\mathrm{Frequency}\;f_{t}^{}$ [kHz]	Mass m [g]	Length L * [mm]	Width b * [mm]	Hight t * [mm]	Young Modulus E [GPa]	Shear Modulus G [GPa]	Poisson’s Ratio μ
Steel									
S1/1	2.335	7.973	236	150	20	10	205.524	81.817	0.256
S1/2	2.335	7.973	236	150	20	10	205.524	81.817	0.256
S1/3	2.335	7.973	236	150	20	10	205.524	81.817	0.256
S2/1	2.335	7.964	236	150	20	10	205.524	81.632	0.259
S2/2	2.335	7.964	236	150	20	10	205.524	81.632	0.259
S2/3	2.335	7.964	236	150	20	10	205.524	81.632	0.259
S3/1	2.336	7.967	236	150	20	10	205.700	81.694	0.259
S3/2	2.336	7.967	236	150	20	10	205.700	81.694	0.259
S3/3	2.336	7.967	236	150	20	10	205.700	81.694	0.259
**Average**							**205.583**	**81.714**	**0.258**
Aluminum									
S1/1	2.337	7.653	85	150	20	10	73.517	27.079	0.357
S1/2	2.337	7.653	85	150	20	10	73.517	27.079	0.357
S1/3	2.337	7.653	85	150	20	10	73.517	27.079	0.357
S2/1	2.335	7.654	85	150	20	10	73.517	27.079	0.357
S2/2	2.335	7.654	85	150	20	10	73.517	27.079	0.357
S2/3	2.335	7.654	85	150	20	10	73.517	27.093	0.357
S3/1	2.336	7.649	85	150	20	10	74.087	27.093	0.367
S3/2	2.336	7.649	85	150	20	10	74.087	27.093	0.367
S3/3	2.336	7.649	85	150	20	10	74.087	27.093	0.367
**Average**							**74.089**	**27.143**	**0.365**
Brass									
S1/1	1.564	5.067	254	150	20	10	99.239	35.565	0.395
S1/2	1.564	5.066	254	150	20	10	99.239	35.551	0.396
S1/3	1.564	5.066	254	150	20	10	99.239	35.551	0.396
S2/1	1.566	5.064	254	150	20	10	99.493	35.523	0.400
S2/2	1.572	5.066	254	150	20	10	100.257	35.551	0.410
S2/3	1.572	5.066	254	150	20	10	100.257	35.551	0.410
S3/1	1.564	5.061	254	150	20	10	99.239	35.481	0.398
S3/2	1.564	5.061	254	150	20	10	99.239	35.481	0.398
S3/3	1.564	5.066	254	150	20	10	99.239	35.551	0.396
**Average**							**99.494**	**35.534**	**0.400**

* the sample dimensions were strictly controlled during the manufacturing process with ±0.01 mm dimensional tolerance for the test purposes.

**Table 5:** IET measurement vs. manufacturer data.

Density [kg/cm^3^]	Electr. Conductivity [MS/m]	Modulus of Elasticity [GPa]	Shear Modulus [GPa]	Poisson’s Ratio
Structural steel (EN S235)	Manufacturers data	205–2010 *	80–82 *	0.25–0.30 *
	Calculated data	≈206	≈82	≈0.26
Aluminium alloy (EN AW-2017A)	Manufacturers data	72–74 *	27–28 *	0.34–0.37 *
	Calculated data	≈74	≈27	≈0.36
Brass alloy (EN CW617N)	Manufacturers data	95–125 *	35–44 *	0.34–0.40 *
	Calculated data	≈99	≈36	≈0.4

* Depending on the manufacturer, the alloy composition, heat treatment, the presence of impurities, and the manufacturing standard used.

## Data Availability

Not applicable.

## References

[1] Scislo L., Guinchard M. Non-Invasive Measurements of Ultra-Lightweight Composite Materials Using Laser Doppler Vibrometry System. Proceedings of the 26th International Congress on Sound and Vibration.

[2] Wiyanto H., Makarim C.A., Gondokusumo O., Siregar J.P., Irawan A.P., Cionita T., Najid N. (2022). Determining Concrete Structure Condition Rating Based on Concrete Compressive Strength. Buildings.

[3] Sun X., Gu J., Tang S., Li J. (2018). Research Progress of Visual Inspection Technology of Steel Products—A Review. Appl. Sci..

[4] Yang G., Liu K., Zhang J., Zhao B., Zhao Z., Chen X., Chen B.M. (2022). Datasets and Processing Methods for Boosting Visual Inspection of Civil Infrastructure: A Comprehensive Review and Algorithm Comparison for Crack Classification, Segmentation, and Detection. Constr. Build. Mater..

[5] Zhang X., Yan Y.H., Chen W.H., Chen J.J. (2012). Image Fusion Method for Strip Steel Surface Detect Based on Bandelet-PCNN. Adv. Mater. Res..

[6] Falorca J.F., Miraldes J.P.N.D., Lanzinha J.C.G. (2021). New trends in visual inspection of buildings and structures: Study for the use of drones. Open Eng..

[7] Dupont Q.F.M., Chua D.K.H., Tashrif A., Abbott E.L.S. (2017). Potential Applications of UAV along the Construction’s Value Chain. Procedia Eng..

[8] Kong X., Li J. (2018). Vision-Based Fatigue Crack Detection of Steel Structures Using Video Feature Tracking. Comput. Civ. Infrastruct. Eng..

[9] Lado-Roigé R., Font-Moré J., Pérez M.A. (2023). Learning-Based Video Motion Magnification Approach for Vibration-Based Damage Detection. Measurement.

[10] Rodríguez-Mariscal J., Canivell J., Solís M. (2021). Evaluating the Performance of Sonic and Ultrasonic Tests for the Inspection of Rammed Earth Constructions. Constr. Build. Mater..

[11] du Plessis A., Boshoff W.P. (2019). A Review of X-ray Computed Tomography of Concrete and Asphalt Construction Materials. Constr. Build. Mater..

[12] Schabowicz K. (2018). Non-Destructive Testing of Materials in Civil Engineering. Materials.

[13] Sacarea A.I., Oancea G., Parv L. (2021). Magnetic Particle Inspection Optimization Solution within the Frame of NDT 4.0. Processes.

[14] Li L., Yang Y., Cai X., Kang Y. (2020). Investigation on the Formation Mechanism of Crack Indications and the Influences of Related Parameters in Magnetic Particle Inspection. Appl. Sci..

[15] Schabowicz K. (2021). Testing of Materials and Elements in Civil Engineering. Materials.

[16] Michałek J., Sobótka M. (2020). Assessment of Internal Structure of Spun Concrete Using Image Analysis and Physicochemical Methods. Materials.

[17] Saba N., Jawaid M., Sultan M.T.H. (2019). An Overview of Mechanical and Physical Testing of Composite Materials. Mech. Phys. Test..

[18] Zhang J., Guo Z., Liu K. (2022). Mechanical Properties Study of Miniature Steel Specimens Based on the Small Punch Test and Simulation Methods. Materials.

[19] Wu H., Lei H., Chen Y.F., Qiao J. (2019). Comparison on Corrosion Behaviour and Mechanical Properties of Structural Steel Exposed between Urban Industrial Atmosphere and Laboratory Simulated Environment. Constr. Build. Mater..

[20] Liu C., Lv S., Peng X., Zheng J., Yu M. (2020). Analysis and Comparison of Different Impacts of Aging and Loading Frequency on Fatigue Characterization of Asphalt Concrete. J. Mater. Civ. Eng..

[21] Ding J., Cheng L., Chen X., Chen C., Liu K. (2021). A Review on Ultra-High Cycle Fatigue of CFRP. Compos. Struct..

[22] Carlesso D.M., de la Fuente A., Cavalaro S.H.P. (2019). Fatigue of Cracked High Performance Fiber Reinforced Concrete Subjected to Bending. Constr. Build. Mater..

[23] Allen E., Iano J. (2013). Fundamentals of Building Construction: Materials and Methods.

[24] Minor A.M., Dehm G. (2019). Advances in in situ nanomechanical testing. MRS Bull..

[25] Giurgiutiu V. (2022). Stress, Vibration, and Wave Analysis in Aerospace Composites.

[26] Braun S., Simon G., Ewins D.J., Rao S.S. (2002). Encyclopedia of Vibration.

[27] Tognana S., Salgueiro W., Somoza A., Marzocca A. (2010). Measurement of the Young’s Modulus in Particulate Epoxy Composites Using the Impulse Excitation Technique. Mater. Sci. Eng. A.

[28] Popov I., Shitikova M.V. (2020). Impulse Excitation Technique and Its Application for Identification of Material Damping: An Overview. IOP Conf. Ser. Mater. Sci. Eng..

[29] Fukumoto Y., Takewaki I. (2015). Critical Earthquake Input Energy to Connected Building Structures Using Impulse Input. Earthq. Struct..

[30] Scislo L. (2022). High Activity Earthquake Swarm Event Monitoring and Impact Analysis on Underground High Energy Physics Research Facilities. Energies.

[31] Scislo L. (2023). Single-Point and Surface Quality Assessment Algorithm in Continuous Production with the Use of 3D Laser Doppler Scanning Vibrometry System. Sensors.

[32] Roebben G., Bollen B., Brebels A., Van Humbeeck J., Van der Biest O. (1997). Impulse Excitation Apparatus to Measure Resonant Frequencies, Elastic Moduli, and Internal Friction at Room and High Temperature. Rev. Sci. Instrum..

